# Synthesis of no-carrier-added [^188, 189, 191^Pt]cisplatin from a cyclotron produced ^188, 189, 191^PtCl_4_^2−^ complex

**DOI:** 10.1038/s41598-021-87576-2

**Published:** 2021-04-14

**Authors:** Honoka Obata, Katsuyuki Minegishi, Kotaro Nagatsu, Mikako Ogawa, Ming-Rong Zhang

**Affiliations:** 1 Department of Advanced Nuclear Medicine Sciences, Institute for Quantum Medical Science (iQMS), National Institutes for Quantum and Radiological Science and Technology (QST), 4-9-1 Anagawa, Inage-ku, Chiba, 263-8555 Japan; 2 Graduate School of Pharmaceutical Sciences, Hokkaido University, Kita-ku, Sapporo, Hokkaido 060-0812 Japan; 3 Japan Society for the Promotion of Science (JSPS), 5-3-1 Kojimachi, Chiyoda-ku, Tokyo 102-0083 Japan

**Keywords:** Nuclear chemistry, Inorganic chemistry

## Abstract

We developed a novel method for production of no-carrier-added (n.c.a.) [^188, 189, 191^Pt]Pt^II^Cl_4_^2−^ from an Ir target material, and then synthesized n.c.a. [*Pt]cis-[Pt^II^Cl_2_(NH_3_)_2_] ([*Pt]cisplatin) from [*Pt]Pt^II^Cl_4_^2−^. [*Pt]Pt^II^Cl_4_^2−^ was prepared as a synthetic precursor of n.c.a. *Pt complex by a combination of resin extraction and anion-exchange chromatography after the selective reduction of Ir^IV^Cl_6_^2−^ with ascorbic acid. The ligand-substitution reaction of Cl with NH_3_ was promoted by treating n.c.a. [*Pt]Pt^II^Cl_4_^2−^ with excess NH_3_ and heating the reaction mixture, and n.c.a. [*Pt]cisplatin was successfully produced without employing precipitation routes. After this treatment, [*Pt]cisplatin was isolated through preparative HPLC with a radiochemical purity of 99 + % at the end of synthesis (EOS).

## Introduction

Targeted radionuclide therapy (TRT) is a type of radiation therapy in which malignant tissues are internally irradiated with radiopharmaceuticals emitting with *β*^*–*^ray, *α*-ray, or Auger electron (Auger *e*^*-*^). *β*^*–*^rays are the most commonly used in the clinic. Recently, *α*-rays have attracted a great deal of interest because of their high therapeutic efficacy, e.g., ^225^Ac-PSMA-617 for metastatic castration-resistant prostate cancer^1^. Auger *e*^*-*^, the third candidate, are also expected to be used in TRT, and many radiopharmaceuticals labeled with Auger *e*^*-*^ emitters (e.g., ^123,125^I and ^111^In) have been developed^2^. However, the therapeutic efficacy has been modest or low in clinical trials performed to date^3–7^, and the causes and potential solutions remain unexplored.

An Auger *e*^*-*^ is a low-energy electron released following inner-shell excitation, and each excited atom emits multiple Auger *e*^*-*^. The range of Auger *e*^*-*^ is extremely short, 2–500 nm, yielding a high linear energy transfer (LET) of 4–26 keV/µm in the limited nano-scale range^8^. For example, the locally absorbed radiation dose around an ^125^I decay site was estimated to be 1.6 MGy within a radius of 2 nm^9^. The effective range of Auger *e*^*-*^ is smaller than a single cell, suggesting that it is necessary to transport radiopharmaceuticals to intracellular regions that are sensitive to radiation. DNA is expected to be a prime target of Auger *e*^*-*^ therapy^2,10–13^. More double-strand breaks can be induced when an Auger *e*^*-*^ emitter is closer to the DNA^14–16^, suggesting that radiopharmaceuticals must be brought as close as possible to DNA to ensure an efficient interaction between Auger *e*^*-*^ and DNA in the nano-scale range. Therefore, in many radiopharmaceuticals developed to date, Auger-emitting radionuclides of ^123,125^I and ^111^In were labeled to DNA-targeting molecules, e.g., a nucleic acid derivative such as deoxyuridine (UdR)^3,17^, a nuclear localization signal (NLS)^12,18^, or a DNA-binding molecule^19–21^, to ensure their transport to DNA. Although antimetabolites based on nucleic acid derivatives are incorporated into DNA, they are limited for use in TRT treatment due to their unavoidable distribution in the intestinal tract, which is radiosensitive. In almost all drugs, however, Auger *e*^*-*^ emitters labeled to DNA-targeting molecules are either not combined with DNA, or are combined indirectly through an intermediary molecule; consequently, there is expected to be a distance between the DNA and the Auger *e*^*-*^ emitter. Auger *e*^*-*^ emitters directly combined with DNA may induce DNA damage most efficiently, but most radioelements are not combined with DNA by themselves. Radioelements should be labeled to intermediary DNA-targeting molecules when being transported to DNA, and such drug design is unalterable.

Platinum has a natural property that is useful in this context. Many platinum complexes (e.g., cisplatin, carboplatin, and oxaliplatin) have been used as platinum-based antineoplastic drugs, and platinum complexes with appropriate leaving groups can form direct DNA adducts between Pt and nucleobases^22^. ^191^Pt (*T*_1/2_ = 2.80 d, EC = 100%), ^193m^Pt (*T*_1/2_ = 4.33 d, IT = 100%), and ^195m^Pt (*T*_1/2_ = 4.01 d, IT = 100%), summarized in Table 1,^23,24^, are promising candidate radionuclides^25^ that have a suitable half-life and a very high Auger *e*^*-*^ yield, e.g., an average of 32.8 electrons emitted per decay for ^195m^Pt vs. 14.7 electrons for ^111^In^26^. Therefore, platinum complexes labeled with radio-Pt as the center metal allow many Auger *e*^*-*^ to be released very close to DNA, and are therefore appropriate for detailed studies to make sure of the degree of the therapeutic effect by Auger *e*^*-*^. In this work, we focus on cis-[Pt^II^Cl_2_(NH_3_)_2_] (cis-diamminedichloroplatinum (II)), commonly called cisplatin, which can form direct DNA adducts between Pt and nucleobase as an intra-stand cross-link^22^. Cisplatin is a widely used chemotherapeutic agent, and its value is supported by a large number of basic and clinical studies over the years. In the clinic, cisplatin is also used in combination with external radiation because it can increase therapeutic efficacy by causing DNA damage via different routes ^27^. Because radio-Pt–labeled cisplatin acts as both an anticancer agent that can target and chemically damage DNA and an Auger *e*^*-*^ emitter, it is expected to provide a superior therapeutic effect as an in vivo radio-chemotherapy agent.

**Table 1:** Decay characteristics of relevant platinum radionuclides.

	^195m^Pt	^193m^Pt	^191^Pt	^189^Pt	^188^Pt
Half-life	4.01 d	4.33 d	2.83 d	10.87 h	10.2 d
Decay scheme	IT: 100%	IT: 100%	EC: 100%	EC: 100%	EC: 99 + %
*γ*	98.9 keV (11.7%)	135.5 keV (0.11%)	538.9 keV (13.7%)	721.4 keV (7.9%)	187.6 keV (19.1%)
Auger *e*^-^	L: 140% K: 3.3%	L: 55.2% K: 0.64%	L: 106% K: 5.3%	L: 108% K: 5.4%	L: 82% K: 3.6%

Data for ^188, 189, 193 m, 195m^Pt were taken from NuDat 2.8^23^, and data for ^191^Pt were taken from Radionuclide Decay Data^24^.

Contrary to these expectations, however, the production method of no-carrier-added (n.c.a.) radio-Pt remains to be established at a practical level. Although the degree of therapeutic efficacy was reported in previous studies using carrier-added radio-cisplatin with low specific activity (~ MBq/mg)^28,29^, it was doubtful whether the fundamental potential of Auger *e*^*-*^ itself could be detected without being masked by the chemotherapeutic effects of non-radioactive cisplatin carriers. To reveal the therapeutic potential of Auger *e*^*-*^, the DNA-damaging effect of radio-cisplatin needs to be investigated using n.c.a. radio-Pt. Available radio-Pt is commonly produced by a reactor via the ^nat^Pt(n,x)^191,193 m, 195m^Pt reaction, resulting in carrier-added radio-Pt derived from a non-radioactive Pt target material. Although n.c.a. ^191,193m^Pt can be produced by a cyclotron from a target material of iridium (Ir) or osmium (Os), several issues related to the chemical properties of both Ir and Os make it difficult to produce ^191,193m^Pt with high yield and high purity^30–33^. Therefore, we demonstrated the production of n.c.a. ^191^Pt from an Ir target using a cyclotron^34,35^. In this work, we established a procedure for producing n.c.a. *Pt^II^Cl_4_^2−^ as a synthetic precursor of n.c.a. *Pt complex, as well as a method for synthesis of n.c.a. [*Pt]cisplatin from n.c.a. *Pt^II^Cl_4_^2−^. In the experiments for this study, we used mixed ^188,189, 191^Pt (81.7 ± 0.4% of ^189^Pt, 17.6 ± 0.6% of ^191^Pt, and 0.7 ± 0.2% of ^188^Pt at the end of bombardment [EOB]), described as *Pt in the following, because ^188,189^Pt is co-produced along with ^191^Pt from a natural Ir target.

## Materials and methods

### General

Natural Ir powder (99.9%, d_50_ = 22.560 µm [median size]) was purchased from Furuya Metal (Tokyo, Japan), and sodium peroxide (95%) was purchased from Hayashi Pure Chemical Industry (Osaka, Japan). Ascorbic acid injection (500 mg/2 mL) was purchased from Fuso Pharmaceutical Industry (Osaka, Japan). Other chemicals and reagents were purchased from FUJIFILM Wako Pure Chemical (Osaka, Japan), Tokyo Chemical Industry (Tokyo, Japan), Kanto Chemical (Tokyo, Japan), Otsuka Pharmaceutical Factory (Tokyo, Japan), or Sigma-Aldrich (St. Louis, MO, USA), and were used in experiments without further purification. Milli-Q ultrapure water was used for dilution in all experiments.

HPGe *γ*-ray spectrometry was used for radioactivity measurements. The HPGe detector (EGC 15–185-R, Eurisys Measures, Strasbourg, France) was coupled with a 4096 multi-channel analyzer (RZMCA, Laboratory Equipment, Ibaraki, Japan), and calibrated using a mixed (^109^Cd, ^57^Co, ^139^Ce, ^51^Cr, ^85^Sr, ^137^Cs, ^54^Mn, ^88^Y, and ^60^Co) standard source (Japan Radioisotope Association, Tokyo, Japan). The efficiencies of each chemical separation process were defined as the ratio of ^191^Pt radioactivity after separation vs. before separation.

The HPLC system (PU-4080i; MD-4010, Jasco, Tokyo, Japan) was equipped with a 200-µL (analysis) or 1-mL (preparative isolation) sample loop and a radiation detector (US-3000, Universal Giken, Kanagawa, Japan). The analysis of *Pt^II^Cl_4_^2−^ was performed using a Nucleosil SB anionic exchange column (Chemco Plus Scientific, Osaka, Japan) at a flow rate of 1.5 mL/min, and eluted with perchlorate solution (MeCN/H_2_O = 40/60 (v/v) containing 0.1 mol/L NaClO_4_ and 0.04 mol/L HClO_4_). The analysis of [*Pt]cisplatin was performed using a polymer-based aqueous size exclusion chromatography (SEC) column (OHpak SB-804 HQ, Shodex, Showa Denko, Tokyo, Japan) at a flow rate of 1.0 mL/min, eluted with saline solution. Non-radioactive reference samples, K_2_Pt^II^Cl_4_ prepared in 0.1 mol/L HCl (0.5 mg/mL), and non-radioactive cisplatin and transplatin in saline solution (0.5 mg/mL) were also analyzed to identify respective retention times.

### Preparation of n.c.a. *Pt^II^Cl_4_^2−^

**Figure 1:**
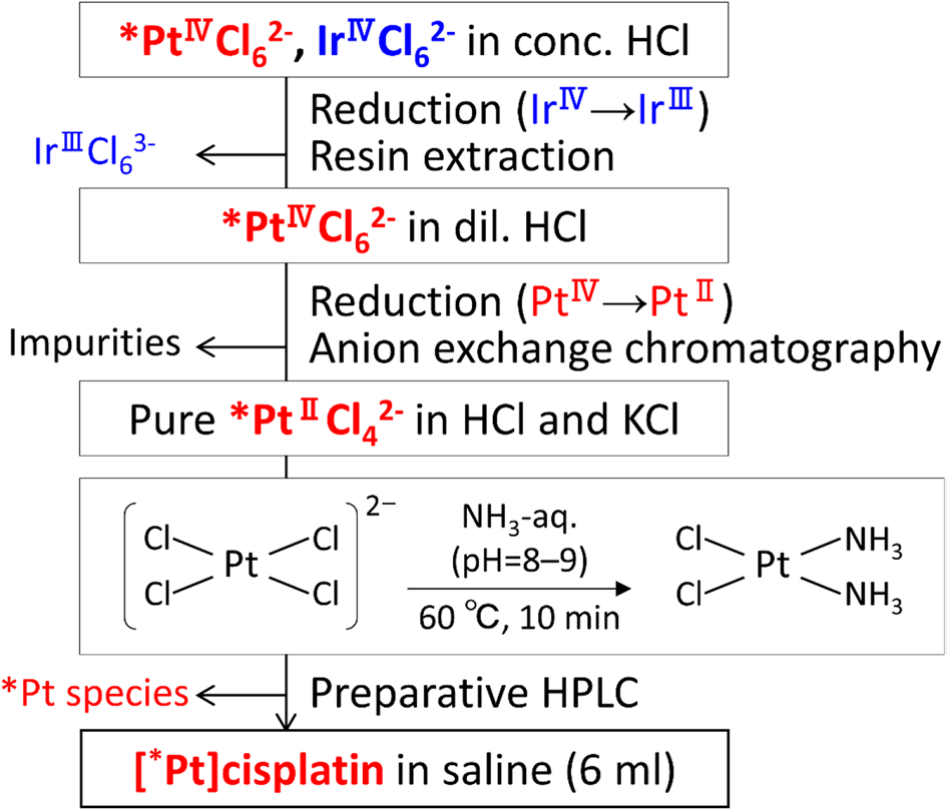
Scheme for preparation of *Pt^II^Cl_4_^2−^ and synthesis of [*Pt]cisplatin.

The preparation scheme is shown in Fig. 1; the details of this scheme are as follows. As described previously^35^, ^188,189, 191^Pt was produced via the ^nat^Ir(p,xn)^188,189, 191^Pt reaction with a 30-MeV proton beam for 2–3 h at a beam current of 9–10 μA, and the irradiated Ir target (Ir: 120 mg, Na_2_O_2_: 98 mg) was dissolved in 6 mol/L HCl (6 mL). After filtering the solution, a stock solution containing mostly Ir^IV^Cl_6_^2−^ with trace amounts of *Pt^IV^Cl_6_^2−^ was prepared. In a typical batch, about 660 (^189^Pt) + 142 (^191^Pt) + 6 (^188^Pt) MBq of *Pt^IV^Cl_6_^2−^ was produced at EOB (81.7 ± 0.4% of ^189^Pt, 17.6 ± 0.6% of ^191^Pt, and 0.7 ± 0.2% of ^188^Pt) and was used in the experiments.

Ascorbic acid, a reducing agent, was added to the filtered solution to selectively reduce Ir^IV^Cl_6_^2−^ (ascorbic acid injection/Ir solution = 0.15/6 [v/v]). During this procedure, the dark reddish-brown solution turned greenish-yellow, as Ir^IV^Cl_6_^2−^ was selectively reduced to Ir^III^Cl_6_^3−^ whereas *Pt^IV^Cl_6_^2−^ remained intact. After the reduction, the mixed solution was loaded into a TBP-resin column made by connecting three TBP-resin cartridges (2 mL cartridge, TrisKem International, Rennes, France), as *Pt^IV^Cl_6_^2−^ was selectively extracted into the resin. The column was rinsed with 3 mol/L HCl (5 mL), and then water (6 mL) was used as an eluting agent. To reduce *Pt^IV^ to *Pt^II^, an ascorbic acid solution (water + ascorbic acid injection = 2 + 8 mL) was added to the collected elution (6 mL), yielding a crude *Pt^II^Cl_4_^2−^ solution. The HCl concentration of this mixed solution was estimated to be < 1 mol/L.

The resultant crude *Pt^II^Cl_4_^2−^ solution (16 mL) was loaded onto a column of QMA (Φ15 × 40 mm, AccellPlus QMA, Waters, Milford, MA, USA) for further purification, and the column was rinsed with 0.01 mol/L HCl (12 mL). An aqueous solution containing 1.5 mol/L HCl and 0.02 mol/L KCl, was used as an eluting agent. The elution was fractionated (2 mL each, f1–12), and the fractions containing *Pt^II^Cl_4_^2−^ (f5–10) were collected based on their radioactivity. The volume of the collected solution was decreased by evaporation, and the *Pt^II^Cl_4_^2−^ product (a precursor of [*Pt]cisplatin) was prepared in HCl and KCl solution (< 300 μL).

### Synthesis of n.c.a. [*Pt]cisplatin

The synthesis scheme is shown in Fig. 1. About 900 μL of 3 mol/L ammonia solution was added to the *Pt^II^Cl_4_^2−^ solution until the pH reached a value of 8–9, as determined using a pH meter (D-72LAB; 9618S-10D, HORIBA, Kyoto, Japan). The mixed solution was heated in hot water at 60 °C for 10 min, and then cooled in ice water. To isolate [*Pt]cisplatin, preparative HPLC was performed using a polymer-based aqueous size exclusion chromatography (SEC) column (OHpak SB-2004, Shodex, Showa Denko, Tokyo, Japan) at a flow rate of 3.0 mL/min, eluted with physiological saline solution.

## Results and discussion

### Preparation of n.c.a. *Pt^II^Cl_4_^2−^

We prepared n.c.a. *Pt^II^Cl_4_^2−^ by a combination of resin extraction and anion exchange chromatography as a precursor for the synthesis of n.c.a. [*Pt]cisplatin. First, *Pt^IV^Cl_6_^2−^ was separated from a bulk Ir target through a TBP-resin column extraction after selectively reducing Ir^IV^Cl_6_^2−^. Our comprehensive survey of reductants revealed that ascorbic acid had the highest selectivity for the reduction of Ir^IV^Cl_6_^2−^ in 6 mol/L HCl. By contrast, *Pt^IV^Cl_6_^2−^ was not reduced at all in 6 mol/L HCl, but was easily reduced in dil. HCl (< 1 mol/L). As a result of the successful selective reduction of Ir^IV^Cl_6_^2−^, only *Pt^IV^Cl_6_^2−^ was extracted from the bulk Ir^III^Cl_6_^3−^ solution onto the extraction column. Ascorbic acid enables the selective reduction of Ir^IV^Cl_6_^2−^ and makes the following separation processes much efficient, compared to acetaldoxime used in our previous study.

In this work, the solid-phase extraction was applied in place of the liquid–liquid extraction because column separation is more suitable for expansion into a remote automatic device for further development. *Pt^IV^Cl_6_^2−^ was extracted onto the TBP-resin column in the presence of HCl, and then quickly eluted with H_2_O. The extraction efficiencies for the recovery of *Pt were above 90% (n = 3, Table 2).

**Table 2:** Chemical separation efficiency of *Pt (n = 3).

	Separation of *Pt from an Ir target	
	Resin extraction (%)	Purification (AEC) (%)
1	91	61
2	93	60
3	90	70

Efficiency contains an uncertainty of 5% in the radioactivity measurement.

While only Ir^IV^Cl_6_^2−^ was reduced by ascorbic acid in 6 mol/L HCl, we found that ascorbic acid is also applicable to reduce *Pt^IV^Cl_6_^2−^ to *Pt^II^Cl_4_^2−^ in dil. HCl (< 1 mol/L). In the reduction of n.c.a. *Pt^IV^Cl_6_^2−^ (< 0.1 nmol), the equivalent amount of reductant is very small, leading to a very low concentration (nmol/L). In that condition, the reducing reaction of n.c.a. *Pt^IV^Cl_6_^2−^ did not proceed by conventional reductants used for Pt^IV^Cl_6_^2−^ underwater (e.g., hydrazine, oxalate, sulfite). Although excess reductants promoted the reduction of n.c.a. *Pt^IV^Cl_6_^2−^ to *Pt^II^Cl_4_^2−^ in a neutralizing solution, undesired hydrolysis or ligand substitution also proceeded and generated unknown species. Therefore, we searched reductants used for n.c.a. *Pt^IV^Cl_6_^2−^ to *Pt^II^Cl_4_^2−^ in HCl, where platinum chloride is more stable, and consequently, ascorbic acid was the most suitable agent in our method for preparing n.c.a. *Pt^II^Cl_4_^2−^.

**Figure 2:**
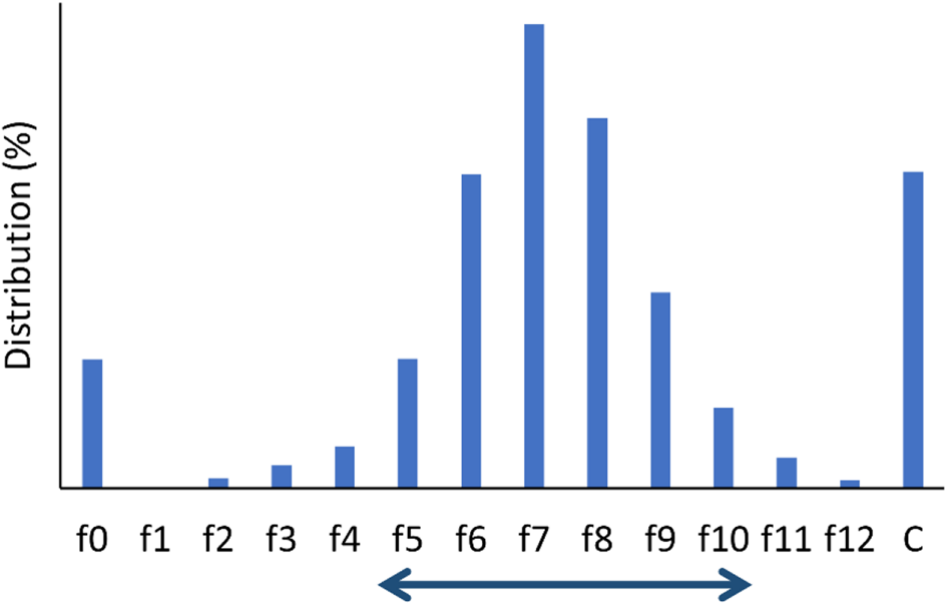
Radiochromatogram obtained during QMA-purification (f0: non-retaining fraction before elution, C: residual on the column). Fractions from f5 to f10 were collected.

After elution from the TBP-resin column, *Pt^IV^Cl_6_^2−^ was reduced by ascorbic acid rapidly in < 1 mol/L HCl. Then, the crude *Pt^II^Cl_4_^2−^ solution was purified by anion exchange chromatography (AEC) with a QMA column. The radiochromatogram obtained during QMA-AEC is shown in Fig. 2. Although *Pt^II^Cl_4_^2−^ was predominantly observed (f2–12), some *Pt^II^Cl_4_^2−^ changed to other complexes, which passed through the column without any interaction (f0) or were strongly retained and remained on the column (C), as shown in Fig. 2. Additionally, the early eluted fractions (f1–4) were removed from the product because they contained impurities derived from ascorbic acid. As a result of these losses, a pure fraction of n.c.a. *Pt^II^Cl_4_^2−^ was isolated at an efficiency of 60–70% (n = 3, Table2). Overall, as summarized in Table 2, the efficiency of the preparation of *Pt^II^Cl_4_^2−^ was nearly constant, and the n.c.a. *Pt^II^Cl_4_^2−^ product was obtained. Furthermore, no organic solvents were used in our method for separation of *Pt^IV^Cl_6_^2−^ from a bulk Ir target, which contributes the green chemistry and reduces the workloads on the quality control.

### Synthesis of n.c.a. [*Pt]cisplatin

**Figure 3:**
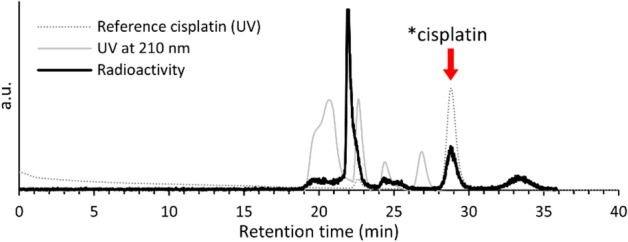
Radiochromatogram obtained during preparative HPLC.

As iodide shows stronger trans effect than chloride, the traditional synthetic scheme of cisplatin involves the conversion of K_2_PtCl_4_ to K_2_PtI_4_^36^. Additionally, bulk cisplatin is commonly produced by forming a crystal precipitate^36,37^. However, it is difficult to precipitate n.c.a. radionuclides (pg–ng), and fewer synthetic steps are suitable to avoid any loss. Therefore, we developed a one-pot radiosynthesis of n.c.a. [*Pt]cisplatin from *Pt^II^Cl_4_^2−^ in solution, and separated it by preparative HPLC. The radiochromatogram obtained during preparative isolation is shown in Fig. 3. [*Pt]cisplatin was detected at a retention time of 28–30 min, in good agreement with the value for non-radioactive cisplatin. The trans isomer (transplatin) was not observed in this radiosynthesis while transplatin is eluted long after cisplatin at 47–49 min (data not shown). The radiochemical yield for [*Pt]cisplatin, defined as the ratio of ^191^Pt radioactivity of isolated [^191^Pt]cisplatin to the total ^191^Pt collected after evaporation, was 5–15%. The low efficiency was due to a decrease in *Pt^II^Cl_4_^2−^ purity during evaporation, in addition to the low synthetic yield of the ligand-substitution reaction between Cl and NH_3_. In HPLC analyses using an anion-exchange column, the peak intensity of *Pt^II^Cl_4_^2−^ decreased with time, whereas an unknown peak that was not retained on the column and a peak of *Pt^IV^Cl_6_^2−^ appeared. The purity of *Pt^II^Cl_4_^2−^ in the evaporated solution was 60% or less, suggesting that n.c.a. *Pt^II^Cl_4_^2−^ is unstable. In support of this observation, the synthesis yield was increased to 30–40% when the collected elution (1 mol/L HCl and 0.5 mol/L KCl) from the QMA column was used immediately without evaporation.

Even when the purity of *Pt^II^Cl_4_^2−^ was not so reduced, the synthetic yield was less than 50%. In the conventional synthetic method for bulk cisplatin, the synthetic yield is around 60% when K_2_[Pt^II^Cl_4_] is treated directly with NH_3_, and an accurate two equivalents of NH_3_ to Pt should be added in order to prevent excess ligand substitutions^37,38^. In this study, we used n.c.a. *Pt^II^Cl_4_^2−^ and treated it with excess NH_3_, probably resulting in low synthetic yield. Nevertheless, it should be noted that heating was essential to promote the ligand substitution reaction between n.c.a. *Pt^II^Cl_4_^2−^ and excess NH_3_. Without heating, about 50% of *Pt^II^Cl_4_^2−^ remained unreacted under a pH value of 9, indicating that both NH_3_ and heat to some degree promoted ligand substitution for ~ 10^–11^ mol of *Pt, which was interesting from the standpoint of radiochemistry. Although it is quite difficult to finely control the stoichiometric balance of NH_3_ and n.c.a. *Pt^II^Cl_4_^2−^, an appropriate NH_3_ concentration may improve the synthesis yield.

Overall, using our method, n.c.a. [*Pt]cisplatin was finally obtained in saline solution (6 mL) from *Pt^IV^Cl_6_^2−^ in a bulk Ir target, and at the end of synthesis (EOS), following a one-day cool-down period after EOB, about 1.29 (^189^Pt) + 1.00 (^191^Pt) + 0.05 (^188^Pt) MBq/mL of [*Pt]cisplatin was available for further biological studies.

### Quality control

**Figure 4:**
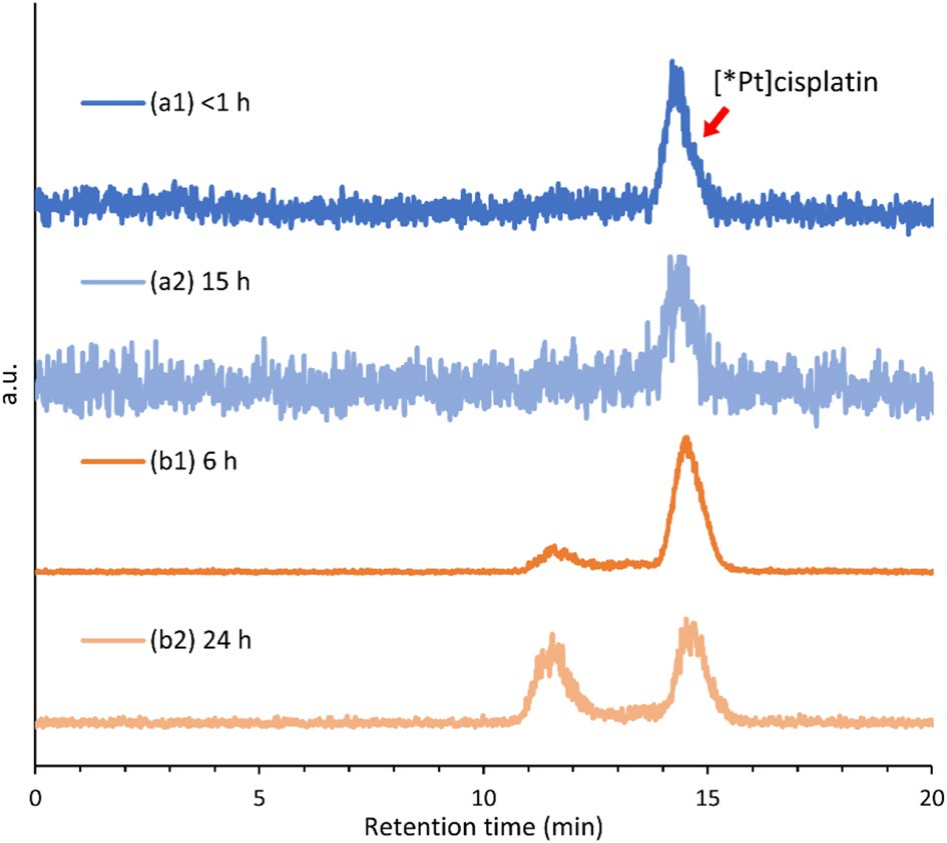
Radiochromatograms of a [*Pt]cisplatin product (**a**) 0.37 (^189^Pt) + 0.31 (^191^Pt) + 0.01 (^188^Pt) MBq/mL at EOS, (**b**) 1.76 (^189^Pt) + 1.40 (^191^Pt) + 0.05 (^188^Pt) MBq/mL at EOS.

We investigated the radiochemical purity and stability of [*Pt]cisplatin by HPLC analyses. As shown in Fig. 4a1, a single peak of [*Pt]cisplatin was observed with a retention time of 14–15 min. The radiochemical purity of n.c.a. [*Pt]cisplatin was 99 + % in the final product. Any impurities were below the detection limit in the chromatogram generated by detecting UV absorption at 250 nm. In the sample with low radioactive concentration shown in Fig. 4a1,2 (0.37 (^189^Pt) + 0.31 (^191^Pt) + 0.01 (^188^Pt) MBq/mL at EOS), [*Pt]cisplatin exhibited good stability in the solution, and radiochemical purity was constant up to 15 h after EOS. By contrast, in the high radioactive concentration shown in Fig. 4b1,2 (1.76 (^189^Pt) + 1.40 (^191^Pt) + 0.05 (^188^Pt) MBq/mL at EOS), [*Pt]cisplatin decomposed as time passed. In this higher concentration, the radiochemical purity decreased to 84% (6 h) and 54% (24 h) after EOS. Although reference transplatin was eluted at 22 min, its peak was not observed in all analyses of [*Pt]cisplatin (data not shown). Therefore, the decomposition product at 11–12 min of Fig. 4b1,2 was not transplatin thermodynamically preferred to cisplatin. We assume that [*Pt]cisplatin decomposed due to hydrolysis or ligand substitution followed by the radiolysis by *γ*-ray, X-ray, and Auger *e*^*-*^ emitted from *Pt.

In HPGe *γ*-ray spectrometry, only ^188,189, 191^Pt were detected, and other coexistent radionuclides in a stock solution (e.g., ^190 g, 192g^Ir) were below the detection limit after purification with QMA-AEC. Due to the overlapping nuclear reaction channels, the product included not only ^191^Pt but also ^188,189^Pt^34^.

## Conclusions

We developed a novel method for production of *Pt^II^Cl_4_^2−^ from an Ir target by employing the selective reduction of Ir^IV^Cl_6_^2−^ with ascorbic acid. Using a combination of resin extraction and AEC, we prepared n.c.a. *Pt^II^Cl_4_^2−^ as a precursor of [*Pt]cisplatin. N.c.a. [*Pt]cisplatin was successfully obtained by treating n.c.a. *Pt^II^Cl_4_^2−^ with excess NH_3_ and heating the reaction mixture. N.c.a. [*Pt]cisplatin was prepared at high radiochemical purities (99 + %), which is useful for evaluating the biological effects of Auger *e*^*-*^ using [*Pt]cisplatin.
